# Telephone Emergency Service 142 (TelefonSeelsorge) during the COVID-19 Pandemic: Cross-Sectional Survey among Counselors in Austria

**DOI:** 10.3390/ijerph18052228

**Published:** 2021-02-24

**Authors:** Elke Humer, Christoph Pieh, Thomas Probst, Ida-Maria Kisler, Wolfgang Schimböck, Petra Schadenhofer

**Affiliations:** 1 Department for Psychotherapy and Biopsychosocial Health, Danube University Krems, 3500 Krems, Austria; christoph.pieh@donau-uni.ac.at (C.P.); thomas.probst@donau-uni.ac.at (T.P.); 2 ABILE-Viktor Frankl Education Austria, 3390 Melk, Austria; dr.i.kisler@gmail.com (I.-M.K.); wolfgang.schimboeck@liwest.at (W.S.); p.schadenhofer@kirche.at (P.S.); 3 Telephone Emergency Service—Lower Austria (TelefonSeelsorge NÖ), Diocese St. Pölten, 3100 St. Pölten, Austria

**Keywords:** COVID-19, counseling, helpline, loneliness, stress, volunteers, well-being

## Abstract

Telephone emergency services play an important role in providing low-threshold, anonymous crisis intervention free of cost. The current study aims to examine the mental well-being and perceived stress level of counselors as well as the main topics of helpline callers during the COVID-19 pandemic in Austria. In the current study, 374 counselors were recruited within the Austrian nationwide organization TelefonSeelsorge during the second wave of COVID-19 infection in Austria. The mental well-being (WHO-5) and perceived stress-level (PSS-10) were assessed and counselors were asked about the frequency of different topics thematized by callers and changes compared to pre-pandemic times. Compared to a reference group of the Austrian general population, counselors experienced less stress (13.22 vs. 16.42) and higher mental well-being (66.26 vs. 57.36; *p* < 0.001). The most frequent topics during the second wave of the COVID-19 pandemic in Austria were loneliness and mental health. More calls were registered in 2020 compared to 2019 and especially the topics loneliness, mental health, professional activities and relationships were reported to be thematized more often during the COVID-19 pandemic compared to the time before (*p* < 0.001). The results contribute to an understanding of the impact of the COVID-19 pandemic on telephone crisis intervention.

## 1. Introduction

The Coronavirus disease 2019 (COVID-19) pandemic and the governmental restrictions to combat the rapid spread of the virus influence almost all aspects of life. In addition to the direct impact of the virus on physical health, substantial detrimental economic impacts, increasing anxiety due to the risk of infection, social isolation, confusing media activity, insecurity and the limited capacity of the healthcare system have to be taken into account [1,2]. 

Previous studies highlight an increase in mental disturbances during the COVID-19 pandemic, such as increased depressive, anxiety and insomnia symptoms [3,4]. In the Austrian general population, a prevalence of 21% for depression, 19% for anxiety and 16% for insomnia were observed during the first COVID-19 lockdown in spring 2020 [5]. These high prevalence rates remained at this elevated level even six months after the end of the lockdown, indicating that the detrimental health consequences of the COVID-19 pandemic persisted several months after its outbreak and the end of the lockdown measures, respectively [6]. Also, forefront healthcare workers reported perception of the detrimental impact of COVID-19 on mental health [7,8]. A previous study conducted on psychotherapists during the first lockdown of the COVID-19 pandemic in Austria revealed a higher stress level compared to a representative German sample [9]. However, the mental well-being and stress level of volunteer helpers—such as counselors of telephone emergency services—have not been evaluated so far.

Overall, an increased need for mental health care not only among people with pre-existing mental health disorders but also among people without pre-existing mental health disorders is expected due to the COVID-19 pandemic [10,11]. There is a range of access barriers to supporting professional mental health care in the form of psychotherapy, because of not being able to afford the financial costs, difficulties experienced in taking time off work, logistic barriers, stigmatizing beliefs and the expectation that mental health problems could or should be managed by oneself independently [12,13,14]. Therefore, readily accessible crisis intervention strategies—such as nonclinical telephone crisis support services—provide essential interventions for individuals affected by the crisis, especially in terms of suicide prevention [15]. As low-threshold support services provide an opportunity to address acute crises, they might even gain importance during as well as in the aftermath of the COVID-19 pandemic.

Telephone emergency services (TES) play an important role in crisis intervention and suicide prevention around the world [16]. The International Federation of Telephone Emergency Services (IFOTES) was founded in 1967 to offer emotional support, immediately accessible to any person in a state of psychological crisis. The IFOTES brings together 31 associations and national federations from 24 countries; one of them is the Austrian TES named TelefonSeelsorge, which was already presented in IFOTES in 1967. TES offer confidential, strictly anonymous, free-of-charge support that is available 24/7 for all individuals in any state of emotional crisis. In Austria, 153,320 calls with at least 30 s duration were registered by the “TES 142” in 2019 and 170,628 in 2020. Trained volunteer counselors provide the TES-service. In this context, previous reports from Germany [16], the United Kingdom [17], Australia [18] and North America [19] revealed that mental and physical health problems, loneliness, romantic and family relationships, as well as dying and death were the most frequent topics. However, whether the COVID-19 pandemic is associated with changes in topics of helpline callers has not been assessed so far. 

Furthermore, several studies addressed mental well-being in the general population, in individuals with pre-existing psychiatric disorders, as well as in forefront healthcare workers, as summarized above. Also in Austria, mental health in the general population as well as in psychotherapists has been assessed before [5,6,9]. However, the mental well-being and subjective stress level of voluntary counselors of TES have not been evaluated so far. Research suggests that helpers who experience increased psychological distress are unable to respond optimally and to deliver optimal and adequate care [15,20]. Therefore, it is important to elucidate whether TES-counselors need to foster their mental hygiene during the COVID-19 pandemic, to ensure optimal low-threshold crisis intervention during this state of a public health emergency.

In Austria, the first COVID-19 cases were reported on 25 February 2020. Thereupon, the Austrian government introduced obligatory COVID-19 lockdown measures on 16 March 2020 lasting until 30 April 2020. During this first wave, daily confirmed cases peaked during the first COVID-19 lockdown with >1000 confirmed cases per day at the end of March. With the end of the lockdown, daily COVID-19 cases decreased and remained at a low level (<100 cases/day) until the end of June 2020 [21]. The decrease in the number of confirmed COVID-19 cases after the lockdown was accompanied by decreased movement restrictions and allowed traveling to countries with low numbers of COVID-19 cases. From July to November 2020, daily COVID-19 cases started to increase again with a peak on 13 November 2020 with >10,000 confirmed cases per day. To combat the rapid spread of the virus during this second wave, the government introduced a second obligatory COVID-19 lockdown from 17 November until 6 December 2020. Thereafter the strict lockdown measures were relaxed including the reopening of shops beyond basic services to allow for Christmas shopping and limited family gatherings around the Christmas holidays. To lower the number of newly confirmed cases further and to reduce the pressure on hospitals, a third strict COVID-19 lockdown went into effect on 26 December 2020 which was initially planned to end on 24 January 2021 but was prolonged until 7 February 2021. The nationwide curfews entailed restrictions in movement and activities with several exceptions. These exceptions included addressing immediate danger, meeting necessary basic needs of daily life, fulfilling work responsibilities, assistance for people in need and outdoor activities only with the people from the same household, with a safe distance between people. 

The current study aims to examine the mental well-being and perceived stress level of counselors as well as the main topics of helpline callers during the COVID-19 pandemic in Austria. The following research questions (RQs) were addressed:

RQ 1: How manifested are well-being and stress-level in counselors of the TES during the second wave of the COVID-19 pandemic in Austria?

RQ 2: Which topics are thematized by callers of the TES during the second wave of the COVID-19 pandemic in Austria?

RQ 3: Are there changes in the topics thematized by callers of the TES during the COVID-19 pandemic in Austria as compared to pre-pandemic times?

## 2. Materials and Methods

### 2.1. Study Design

To investigate the research questions, a quantitative cross-sectional study was carried out in the form of an online survey using the platform REDCap [22,23]. The information about the survey including the link was sent by “TelefonSeelsorge Österreich” to their counselors (*n* = 856) in December 2020. The survey was open from 18 December 2020 to 24 January 2021 and comprised 73 items in total. In total, 374 counselors completed the survey (response rate = 43.7%).

Counselors’ participation was voluntary, without incentives. Participants had to agree to the data declaration to start the survey (electronic informed consent). The principles outlined by the Declaration of Helsinki were followed and the ethics committee of the Danube University Krems (Austria) approved the study (protocol code: EK GZ 35/2018-2021).

### 2.2. Measures

Counselors reported their gender, age, year that they started to work for TES, average hours working for TES per month, federal state, as well as their educational level.

Well-being was assessed with the WHO-5 questionnaire, which is a short inventory with good psychometric properties [24,25]. The WHO-5 measures well-being over the past two weeks with five self-rating items such as “Over the past 2 weeks I have felt cheerful and in good spirits” or “Over the past 2 weeks I have woke up feeling fresh and rested”. The items were rated on a six-point Likert scale from 0 = “at no time” to 5 = “all of the time”, yielding a maximum score of 25. Higher scores indicate higher well-being. Scales of the raw scores were multiplied by four, as measures of health-related quality of life are conventionally translated into a percentage scale from 0 (absence of well-being) to 100 (maximal well-being) [25]. Cronbach’s alpha for the WHO-5 was 0.82 in our sample. From 21 December 2020 to 5 January 2021, we conducted a survey in a representative sample of the Austrian general population with *n* = 1505 participants, showing a mean (M) of 57.36 (SD = 23.16) for the WHO-5 [26].

The counselors’ stress levels were assessed with the Perceived Stress Scale-10 (PSS-10) [27]. The PSS-10 measures the subjective stress level experienced in the last month with 10 items asking about feelings and thoughts during the last month, such as “How often have you felt nervous and stressed” or “How often have you found that you could not cope with all the things that you had to do”. Each item was rated on a five-point Likert scale from 0 = “never” to 4 = “very often”. The items 4, 5, 7 and 8 are positively worded and therefore reverse scored. The total score of the PSS-10 is obtained by summing up all scores, with higher scores indicating higher stress levels. In our sample, Cronbach’s alpha was 0.81. The stress-level of employed persons in a representative German sample was M = 12.32 (SD = 6.30) [28]. Moreover, a recent study in Austrian psychotherapists conducted during the first COVID-19 lockdown in Austria observed a stress level of M = 13.27 (SD = 5.85) [9]. In our study conducted in a representative sample of the Austrian general population from 21 December 2020 to 5 January 2021, we observed an M = 16.42 (SD = 7.60) for the PSS-10 [26]. 

Participants were asked to rate the frequency of thematization of the following 15 topics by callers from “1 = never” to “6 = always” on a six-point Likert scale: 1. Corona, 2. Loneliness, 3. Physical health, 4. Mental health (including depression, anxiety, obsessive-compulsive disorder, self-harming behavior, confusion, worry about mentally sick people), 5. Life events/crises; death/grief; a stroke of fate, 6. Violence/abuse (violence against adults, violence against/abuse of children), 7. Romantic and family relationships; Parenting, generation conflicts, partner search, 8. Professional activities (education, training, job search, unemployment, problems in education (e.g., bullying), material problems (i.e., housing, finances), 9. Information/technical advice, 10. Addiction, 11. Suicide (suicidality/suicide, worry to suicide-prone), 12. Meaning/belief/values, 13. Sexuality, 14. Pregnancy, 15. Refugees (worry about refugees, fear of problems by refugees, “Islamization”, social abuse).

All counselors who already worked at TES before 2020 were asked to rate whether the frequency of topics 2–15 changed due to the COVID-19 pandemic on a five-point Likert scale from “−2 = significantly less” to “+2 = significantly more”. 

### 2.3. Statistics

The IBM SPSS (IBM Corporation, Armonk, NY, USA) Statistics 26 software program was used for statistical analyses. Descriptive statistics were conducted to describe the sociodemographic characteristics. 

Statistics for RQ1: *T*-tests were computed to assess whether mental well-being or perceived stress level in Austrian TES counselors differed from the general population in Austria during the second wave of the COVID-19 pandemic.

Statistics for RQ2: To assess whether the frequency of the different topics differs during the COVID-19 pandemic in Austria linear multilevel models were conducted. The model had two-levels (repeated measures nested within individuals) with the frequency as the outcome variable (rating from 1 to 6) and the topic as a fixed effect. The analysis was performed with the full maximum likelihood method. As a random term, the random intercept was included. Bonferroni corrections were applied for pairwise posthoc tests.

Statistics for RQ3: One-sample *t*-tests were carried out for each of the 14 topics to assess whether they were thematized more or less often than before COVID-19 by comparing the mean to “0”, referring to “no change”. Bonferroni-correction for multiple comparisons was applied for results interpretation of RQ2, considering *p* < 0.0036 as significant (*p* < 0.05/14 *t*-tests). 

All statistical tests were performed two-tailed and the significance level was set to *p* < 0.05 before Bonferroni-correction. We report effect sizes using Hedge’s g with 95% CIs. 

## 3. Results

### 3.1. Participants

In total, 374 Austrian counselors of the TES participated in the online survey. Counselors were on average M = 57.65 (SD = 12.43) years old and were on average M = 8.40 (SD = 8.56) years in the profession. The average working time at the TES was M = 15.58 (SD = 14.16) hours per month. Sociodemographic characteristics are summarized in Table 1.

### 3.2. Results for RQ1

For perceived well-being an average score of M = 66.26 (SD = 16.64) was measured, differing significantly from the M = 57.36 (SD = 23.16) assessed in the Austrian general population during the second COVID-19 wave (T(1, 373) = 10.337; *p* < 0.001). The effect size was moderate, Hedge’s g = 0.40, 95% CI = 0.29, 0.52. 

The counselors stress-level averaged M = 13.22 (SD = 5.20), being significantly lower compared to M = 16.42 (SD = 7.60) measured in the Austrian general population during the second COVID-19 wave (T(1, 373) = −11.891; *p* < 0.001). The effect size was moderate, Hedge’s g = −0.45, 95% CI = −0.56, −0.33. However, no difference in stress level was observed compared to a study conducted in Austrian psychotherapists during the first COVID-19 lockdown in Austria, which observed a stress level of M = 13.27 (SD = 5.85) [9] (T(1, 373) = −0.169; *p* = 0.866). Compared to the stress-level of employed persons in a representative German sample assessed prior to the COVID-19 pandemic (M = 12.32, SD = 6.30; *n* = 1332) [28] a higher stress level was observed (T(1, 373) = 3.366; *p* = 0.001). The effect size was small, Hedge’s g = 0.15, 95% CI = 0.03, 0.26.

### 3.3. Results for RQ2

**Figure 1:**
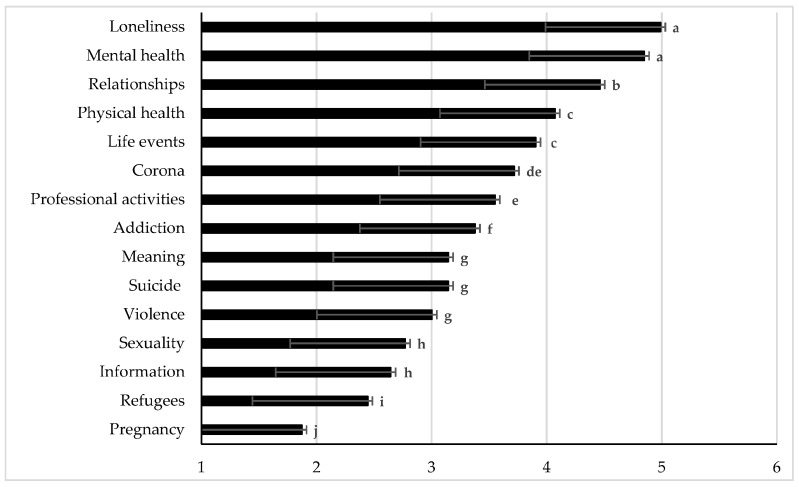
Counselors’ rating of the frequency of topics thematized by helpline callers during the second wave of COVID-19 infection in Austria (*n* = 374). Note: Counselors rated the frequency of each topic on a six-point scale from 1 = “never” to 6 = “always”. Data are shown as least square means (LSM) ± standard error of the means. a,b,c,d,e,f,g,h,i,j Different superscripts indicate differences among LSM of the topics at *p* < 0.05 after Bonferroni-correction.

Significant differences regarding the frequency of different topics during the COVID-19 pandemic were observed (F(14, 5236) = 537.88, *p* < 0.001) as depicted in Figure 1. Loneliness and mental health were the main topics of helpline callers during the second wave of the COVID-19 infection in Austria, differing significantly from all other topics (*p* < 0.001). Also, relationships, physical health and live events were thematized frequently, followed by COVID-19, professional activities, addiction, meaning, suicide and violence. The topics of sexuality, information, refugees and pregnancy were less frequent topics.

### 3.4. Results for RQ3

**Figure 2:**
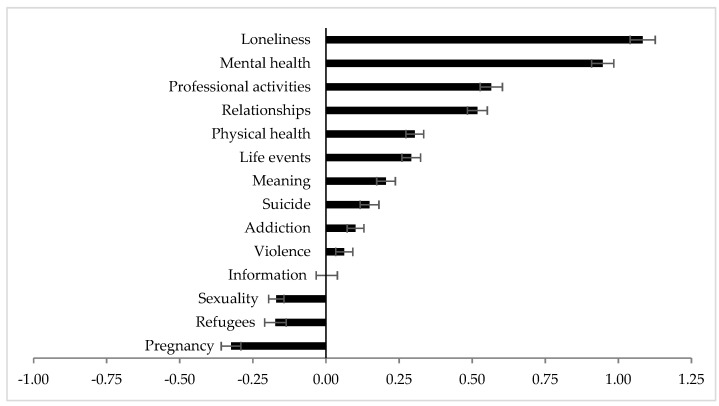
Rating of the change of the frequency of topics thematized by helpline callers during the COVID-19 pandemic as compared to the times before COVID-19 by Austrian counselors (*n* = 336). Note: Counselors who already worked at the telephone emergency service before 2020 were asked to rate whether the frequency of the listed topics changed due to the COVID-19 pandemic on a five-point scale from −2 = “significantly less” to 2 = “significantly more”, with 0 referring to “no change”. Data are shown as least square means (LSM) ± standard error of the means.

As depicted in Figure 2 the 336 counselors who started to work at TES before 2020 reported that the topics loneliness, mental health, professional activities, relationships, physical health, life events, meaning, suicide and addiction were thematized more often by helpline callers during COVID-19 as compared to the time before (*p* < 0.001). For refugees, sexuality and pregnancy the opposite was observed (*p* < 0.001). No difference was reported for violence (*p* = 0.033) and information (*p* = 0.935).

## 4. Discussion

The major aim of this study was to evaluate perceived mental well-being in TES counselors during the second wave of the COVID-19 pandemic in Austria. Results suggest higher mental well-being and less psychological distress in TES counselors compared to the general population during the second COVID-19 wave. 

A previous systematic review suggests that telephone crisis support workers experience elevated symptoms of distress, burnout and psychiatric disorders [15]. These contradictory findings might be due to methodological differences, as only one of the seven studies included in the aforementioned review had a control group, whereas results in our study were compared to measures conducted in a representative sample of the general population. One explanation for the better mental health status of TES counselors observed in the current study might be the higher age of TES counselors (M = 57.65, SD = 12.43) compared to the survey conducted in the Austrian general population during the second COVID-19 wave (M = 45.45, SD = 15.61), as mental well-being is less impaired with increasing age [5]. However, even when comparing individuals aged 60 or older, higher mental well-being (T(1, 193) = 3.180; *p* = 0.002) and less perceived stress (T(1, 193) = −3.886; *p* < 0.001) were observed in TES counselors (*n* = 194; WHO-5: M = 68.93, SD = 19.98; PSS-10: M = 12.03, SD = 5.00) compared to the general population (*n* = 313; WHO-5: M = 65.28, SD = 20.60; PSS-10: M = 13.42, SD = 6.85). This result is supported by previous studies, highlighting that the elderly who engage in voluntary work show higher levels of psychological well-being [29]. Therefore, results suggest that TES counselors were less prone to experience psychological distress during the COVID-19 pandemic as compared to the general population. In agreement, volunteer work has been reported to enhance well-being [30,31], with individuals who volunteer regularly experiencing their lives as more worthwhile [31]. However, people with better mental health are more likely to be willing to invest time in volunteer work [30]. Therefore, volunteer work seems not only to be beneficial for society, but also for the individuals who perform it. 

The second aim of this study was to evaluate whether the COVID-19 pandemic was associated with changes in topics of helpline callers on “TES 142”. Overall, not only the quantity of helpline calls increased (from 153,320 in 2019 to 170,628 in 2020) but also the topics thematized by callers. Results show that especially the topics loneliness, mental health, professional activities and relationships were thematized more often as compared to pre-pandemic times, whereas the opposite was observed for the topics pregnancy, refugees and sexuality. For violence/abuse and information, no difference was reported. Overall, the topics of loneliness and mental health were reported to be the main topics of callers seeking help in TES. 

The finding that the frequency of the predominant topics loneliness and mental health increased significantly under COVID-19 conditions is supported by previous studies highlighting a strong increase in mental health issues during the pandemic [3,5,6]. In free text questions about what could support the work at TES, the most frequent comments by counselors were additional advanced training in the field of mental disorders and additional supervision (data not shown), further highlighting the increased burden of mental disorders among the helpline callers. Isolation and a decrease of social contacts were reported to range among the most important risk factors for mental distress people are exposed to during the COVID-19 situation [32]. Another study in Austria showed that loneliness in combination with perceived-stress during the first lockdown were risk factors for depression after lockdown [33]. A higher risk of domestic violence was also reported and increased incidents of domestic violence following lockdown measures were reported previously for several countries around the world [34,35]. In our study, TES counselors reported no change in the frequency of the topic violence during COVID-19 as compared to the times before. However, from these data, it cannot be concluded that domestic violence was not affected by the COVID-19 situation in Austria. One reason for the not significant increase might be that in Austria there are several helplines especially focusing on violence available (e.g., such as “Frauenhelpline gegen Gewalt” (Women’s helpline against violence)). Therefore, it is likely that people seeking help in situations of violence and abuse would primarily call these hotlines. Indeed, official data from the Austrian Federal Ministry of the Interior report an increase in actual incidents of domestic violence with police operation during the COVID-19 lockdown [36]. 

The major limitation of the present study is the cross-sectional design, which implies that there might be a recall bias regarding the ratings of the frequency of topics during COVID-19 as compared to the times before the pandemic. Moreover, we cannot say whether counselors’ well-being or stress-level changed during COVID-19 as compared to the time before. As multiple measurement points in a longitudinal design would have had more advantages, we are planning to repeat this survey after the acute phase of the COVID-19 pandemic (presumably in summer 2021). It should also be kept in mind that solely volunteers’ self-reports were analyzed and no objective data. Stress-level, for example, was not completed by more objectively quantifiable biological measurements such as cortisol analyses [37]. However, in online surveys, such analyses are not easily feasible. Also, only counseling via telephone was part of the survey, whereas other formats of the TES (i.e., e-mail and chat) were not accounted for. Furthermore, the results might not be representative for the counselors not participating in the online survey as the survey was conducted only online. Comparisons with other countries with other social support systems and countries which were differently affected by the COVID-19 pandemic would be interesting. Since the study was conducted in Austria, results might only apply to countries with similar mental and social support systems.

## 5. Conclusions

Overall, results suggest that helpline counselor work provided by the Austrian telephone emergency service “TelefonSeelsorge 142” might beneficially affect mental well-being during the COVID-19 pandemic. Moreover, during the COVID-19 pandemic, the topics “loneliness” and “mental health” were not only reported to be the main topics of helpline callers but also to be significantly more often thematized than before the pandemic. This highlights the need for the provision of low-threshold mental healthcare services during and in the aftermath of the COVID-19 pandemic. Furthermore, helpline counselors might benefit from additional advanced training in the field of mental disorders and supervision to deal with the increased burden of mental disorders among the helpline callers.

## Figures and Tables

**Table 1:** Sociodemographic characteristics of the sample.

Characteristics	*n*	%
Gender		
Female	299	79.9
Male	75	20.1
Federal state		
Burgenland	19	5.1
Lower Austria	40	10.7
Vienna	63	16.8
Carinthia	37	9.9
Styria	38	10.2
Upper Austria	41	11.0
Salzburg	69	18.4
Tyrol	28	7.5
Vorarlberg	39	10.4
Education		
Secondary school	7	1.9
Apprenticeship	33	8.8
Vocational secondary school	69	18.4
High School	82	21.9
University	183	48.9

## Data Availability

The raw data supporting the conclusion of this article will be made available by the authors upon reasonable request.
